# Urinary Tract Infections Caused by Uropathogenic *Escherichia coli*: Mechanisms of Infection and Treatment Options

**DOI:** 10.3390/ijms241310537

**Published:** 2023-06-23

**Authors:** Yang Zhou, Zuying Zhou, Lin Zheng, Zipeng Gong, Yueting Li, Yang Jin, Yong Huang, Mingyan Chi

**Affiliations:** 1 State Key Laboratory of Functions and Applications of Medicinal Plants, Guizhou Provincial Key Laboratory of Pharmaceutics, Guizhou Medical University, 4 Beijing Road, Guiyang 550004, China; zy225300@163.com (Y.Z.); zu_ing@163.com (Z.Z.); zhengl2020@126.com (L.Z.); gzp4012607@126.com (Z.G.); nhwslyt@163.com (Y.L.); jinyang4791@163.com (Y.J.); naoko_568@sina.com (M.C.); 2 School of Pharmaceutical Sciences, Guizhou Medical University, 4 Beijing Road, Guiyang 550004, China

**Keywords:** urinary tract infections, uropathogenic *Escherichia coli*, alternative treatment options

## Abstract

Urinary tract infections (UTIs) are common bacterial infections that represent a severe public health problem. They are often caused by *Escherichia coli* (*E. coli*), *Klebsiella pneumoniae* (*K. pneumonia*), *Proteus mirabilis* (*P. mirabilis*), *Enterococcus faecalis* (*E. faecalis*), and *Staphylococcus saprophyticus* (*S. saprophyticus*). Among these, uropathogenic *E. coli* (UPEC) are the most common causative agent in both uncomplicated and complicated UTIs. The adaptive evolution of UPEC has been observed in several ways, including changes in colonization, attachment, invasion, and intracellular replication to invade the urothelium and survive intracellularly. While antibiotic therapy has historically been very successful in controlling UTIs, high recurrence rates and increasing antimicrobial resistance among uropathogens threaten to greatly reduce the efficacy of these treatments. Furthermore, the gradual global emergence of multidrug-resistant UPEC has highlighted the need to further explore its pathogenesis and seek alternative therapeutic and preventative strategies. Therefore, a thorough understanding of the clinical status and pathogenesis of UTIs and the advantages and disadvantages of antibiotics as a conventional treatment option could spark a surge in the search for alternative treatment options, especially vaccines and medicinal plants. Such options targeting multiple pathogenic mechanisms of UPEC are expected to be a focus of UTI management in the future to help combat antibiotic resistance.

## 1. Introduction

Urinary tract infections (UTIs) are the most common bacterial infections and affect one million people globally each year [1,2,3]. Among nosocomial infections, UTIs are second only to lower respiratory tract infections, which represent 24% of such cases in developing countries [4]. A UTI requires the presence of more than 10^5^/mL of bacteria in the urine. The symptoms of UTIs are variable, ranging from no symptoms to a severely unwell patient with a high temperature and, sometimes, secondary bacteremia. UTIs can be divided into three categories: acute pyelonephritis, acute cystitis, and asymptomatic bacteriuria [5]. Pyelonephritis is a type of kidney infection, the symptoms of which include the presence of bacteriuria and pyuria. Cystitis is a type of bladder infection, the symptoms of which include frequent urination, dysuria, suprapubic pain, lower abdominal discomfort, and foul-smelling urine. The vast majority of UTIs present as asymptomatic bacteriuria, primarily affecting the lower urinary tract. In some people, such as pregnant or menopausal women, the elderly, diabetics, prepubertal children, and patients with vesicoureteral reflux, bacteria can ascend to the bladder and kidney, making upper UTIs more likely.

Distressingly, UTIs are not only common but also have a high recurrence rate. Usually, a recurring UTI refers to three or more UTIs within one year, as well as two or more recurrences within six months. The abovementioned population is especially susceptible to chronic recurrent UTIs, which leads to the increased use of antibiotics. Women have a high risk of a UTI over their lifetime, and over 20% experience UTI recurrence in the next 6 months after being cured [6,7]. Recurrent UTIs can be especially frequent during pregnancy and may cause severe adverse consequences for the mother and infant, including preterm birth [8]. Recurrent UTIs are a common problem in the elderly [9]. Menopausal women are more likely to experience recurrent UTIs, as lower levels of estrogen lead to adverse changes in the urogenital epithelium and urogenital microbiome [10]. In addition, UTIs affect nearly one-third of children within one year of birth, and about 30% develop recurrent UTIs [11,12]. Among the causative factors, bladder and bowel dysfunction (BBD) and vesicoureteral reflux (VUR) are risk factors for recurrent UTIs, especially when they occur simultaneously [13,14]. Bacteria re-emerging in the bladder epithelium also cause UTI recurrence [15].

**Figure 1:**
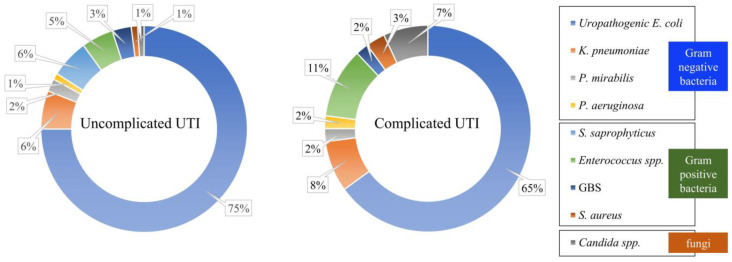
Epidemiology of urinary tract infections (UTIs). UTIs are multispecies; the type of infection can be caused by Gram-negative bacteria, Gram-positive bacteria, or fungi. Among them, uropathogenic *E. coli* (UPEC) are the main pathogenic factor for UTIs, accounting for about 75% of uncomplicated UTI cases and more than half of complicated UTI cases.

UTIs are caused by a wide range of pathogens, including Gram-negative and Gram-positive bacteria, as well as fungi. According to the epidemiology of UTIs (Figure 1) [1,16], uropathogenic *E. coli* (UPEC) are the main pathogenic factor for UTIs, accounting for about 75% of uncomplicated UTI cases; however, less common pathogens, such as *Klebsiella pneumoniae* (*K. pneumonia*), *Staphylococcus saprophyticus* (*S. saprophyticus*), *Enterococcus faecalis* (*E. faecalis*), group B *Streptococcus* (GBS), *Proteus mirabilis* (*P. mirabilis*), *Pseudomonas aeruginosa* (*P. aeruginosa*), *Staphylococcus aureus* (*S. aureus*), and other pathogenic bacteria cause opportunistic UTIs. UPEC UTIs account for more than half of complicated UTI cases [1]. Therefore, in patients suffering from UPEC UTI, maintenance therapy is ensured by antibiotic prophylaxis. Furthermore, UTIs need to be treated by surgery in some cases, especially when UPEC UTIs develop into recurrent UTIs. However, invalid traditional treatments, antibiotic resistance, and the trauma of surgery have made seeking alternative and complementary treatments urgent, with vaccines and medicinal plants showing significant potential. Therefore, UTIs are a major challenge faced by humans worldwide, and ascertaining their causes and finding the most effective treatments are crucial.

“In this review, the UPEC of concern are the most common pathogenic pathogen for UTIs. The properties of UPEC are summarized, including adherence, motility, toxin production, metal acquisition, intracellular bacterial communities (IBCs), and the evasion of host immune defenses, which can help to understand the pathogenesis of UPEC UTI. In addition, the treatment of UTIs is described, including conventional drugs and complementary and alternative medicines, as well as the presence of antibiotic resistance and the great potential of vaccines and medicinal plants. Finally, we present an outlook, with the hope that this paper will draw attention to recent advances in the field of UTI therapeutics”.

## 2. Mechanisms of UPEC UTIs

**Figure 2:**
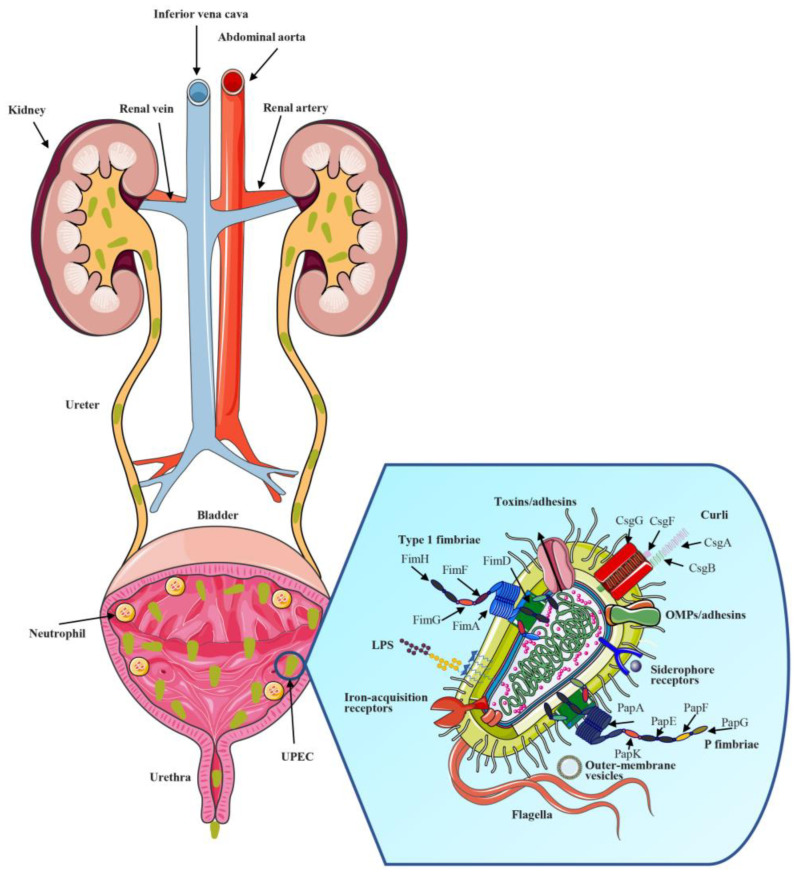
Pathogenesis of UTIs and uropathogenic *E. coli* (UPEC) adhesin structure. UTIs begin when UPEC induce periurethral invasion and colonization. Subsequent UPEC ascension into the bladder and expression of fimbriae and adhesins result in colonization and invasion of host cells and interaction with the defensive system of the bladder epithelium. UPEC produce toxins that induce host cell damage, releasing essential nutrients that promote bacterial survival and ascension to the kidneys. Kidney colonization further leads to bacterial toxin production and host tissue damage. In severe cases, it can lead to the occurrence of diseases such as bacteremia. In the abovementioned process, the structure of UPEC, including type 1 fimbriae, P fimbriae, and other fimbriae and non-fimbrial adhesins, plays an important role in the pathogenic process of UPEC.

The urethra has a self-defense function and usually prevents UTIs. The urethral mucosa and epithelial cells can resist the invasion of pathogenic bacteria, maintaining a balance between the urethra and bacteria. However, when the pathogenicity of bacteria is very strong, or the body suffers from external damage, this balance is disturbed, and the defense function of the body is compromised. Subsequently, a series of inflammatory reactions occur in the urinary tract, such as urethritis, cystitis, and pyelonephritis. Certain factors of UPEC often play an important role in these reactions, such as lipopolysaccharides (LPSs), polysaccharide capsules, flagella, outer-membrane vesicles, fimbriae, curli, non-fimbrial adhesins, outer-membrane proteins (OMPs), and iron-acquisition receptors [16,17]. UPEC are more likely to infect the host from the lower urinary tract. When the impact of UPEC on the body cannot be ignored, the host suffers from a UTI or even more serious disease. Usually, the physiological process of UPEC in the host body can be roughly divided into six steps (Figure 2) [18].

The first step is UPEC-induced periurethral and vaginal invasion and colonization. Various fimbriae and adhesins on the surface of UPEC play a pivotal role in mediating adhesion and colonization. The second step is ascension into the bladder lumen and growth as planktonic cells in the urine. The third step is adherence to the surface and interaction with the defensive system of the bladder epithelium. The fourth step is biofilm formation. UPEC proliferate and accumulate to a certain degree to form the biofilm. The biofilm is important in ensuring that bacterial pathogens can colonize the urinary tract and cause infection. Once the biofilm is formed, it may elude immune defense mechanisms. At the same time, the biofilm also exhibits strong drug resistance, and it is difficult for drugs to penetrate the biofilm, leading to chronic and recurrent UTIs [1,19,20]. The fifth step is invasion and replication via the formation of IBCs in the bladder, where quiescent intracellular reservoirs (QIRs) arise in the underlying urothelium. Bacterial replication in these IBCs can easily reach as high as 10^5^ bacteria per cell. Additionally, bacteria in the IBCs undergo morphological changes, emerge from the infected cell, and enter neighboring cells, which spreads the infection. The sixth step is kidney colonization. UPEC destroy host tissues by releasing toxins, causing upper UTIs, and severe cases may lead to dangerous conditions, including bacteremia, septicemia, urosepsis, and even death [18,21]. These cascade stages occur when UPEC successfully attach to host urothelial cells. In the abovementioned process of UPEC action, adhesins, virulence, IBCs, and other factors have important research significance. In general, the strategies of pathogenesis employed by UPEC include adherence, motility, the acquisition of metals, toxin production, and immune evasion. The bacterial adhesins and their receptors on the host’s cells mediate the processes of bacterial colonization, biofilm formation, replication, secretion of toxins, internalization, and invasion [18,22,23]. In this section, we discuss several of the main factors in UPEC pathogenesis, particularly chaperone–usher fimbrial adhesins, to provide a clearer understanding of the pathogenesis of UPEC UTIs.

### 2.1. UPEC Adhesins

**Figure 3:**
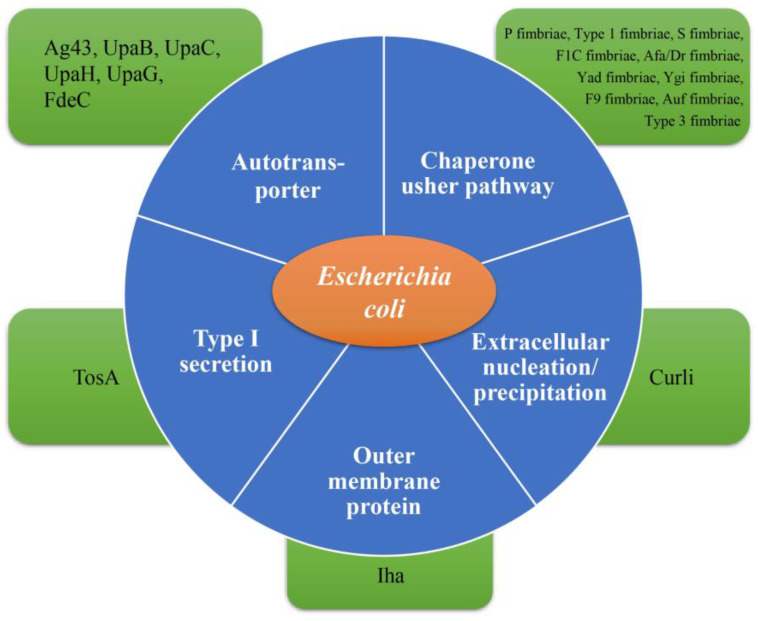
Adhesins of uropathogenic bacteria (orange: organism; blue: assembly pathway; green: adhesins).

Adhesins, a wide range of adhesive proteins assembled by bacteria, can mediate binding to receptors and the colonization of surfaces. Over the past two decades, the whole-genome sequencing of several standard library UPEC strains has revealed the presence of multiple known and putative adhesins in each strain, some of which have been demonstrated to enhance the ability to colonize the urinary tract (Figure 3) [24,25,26,27,28,29]. Among these, adhesive fibers called pili or fimbriae have attracted widespread attention. Fimbriae are long fibers that extend beyond the bacterial capsule. In diverse genera of Gram-negative bacteria, a molecular machine known as the chaperone–usher pathway (CUP) mediates the assembly of fimbriae on the bacterial outer membrane [24]. At the tips of fimbriae, adhesins are thought to play an important role in the adherence of bacteria to host epithelial receptors [25,26,27]. Each sequenced UPEC strain encodes a multitude of CUP operons [28]. At present, several CUP operons, including Auf, Dr, F1C, S, type 9, type 3, type 1, and P fimbriae, have been recognized in UPEC pathotypes (Figure 3), but of those that are broadly conserved among UPEC isolates, only two (type 1 and P fimbriae) have so far been strongly implicated in UTI pathogenesis [29,30,31]. CUP adhesins are known to recognize certain receptors with stereochemical specificity, elucidating causative factors and possible drug mechanisms.

As well-known mannose-sensitive organelles, type 1 fimbriae can generate stable bonds with mannosylated glycoproteins of uroplakins (UPs) [32], as well as N-linked oligosaccharides on *α*3 and *β*1 integrins [33] and the pattern-recognition Toll-like receptor 4 (TLR4) [34], all of which are expressed in the urine bladder to cause successful UTIs. FimH as the tip adhesin of type 1 fimbriae encompasses a lectin domain on the N-terminal head and a fimbrin domain on the C-terminal end, which has a pathogen-associated molecular pattern (PAMP) role [35,36]. As another important fimbrial organelle for UPEC cell adhesion, P fimbriae can bind to *α*-D-galactopyranosyl-1,4-*β*-D-galactopyranoside in the globo-series glycolipids [37], whose tip adhesin is PapG. Therefore, it is important to understand type 1 and P fimbriae and their respective characteristic adhesins.

#### 2.1.1. Type 1 Fimbriae and FimH

**Figure 4:**
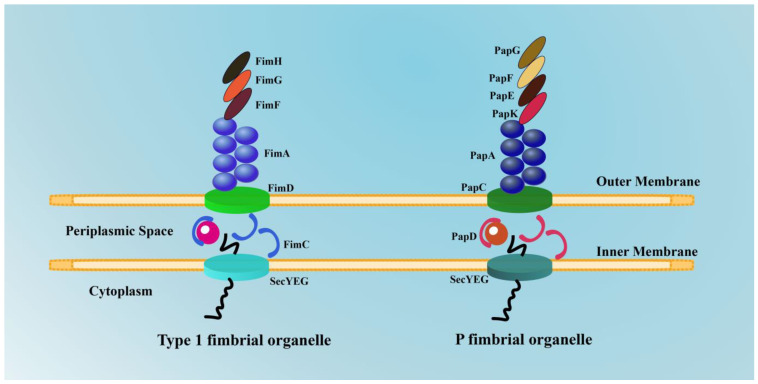
Schematic structure of type 1 fimbrial and P fimbrial organelles in UPEC.

Considerable evidence suggests that type 1 fimbriae are a critical virulence factor in UPEC-derived UTIs in humans [29,30,31,38]. In addition, type 1 fimbriae have also been shown to play a critical role in establishing cystitis in an experimental murine UTI model [39,40], and they are required for UPEC adherence to human urothelial tissue culture cells [41,42]. Many studies have found that nearly five hundred right-handed helical rod-shaped type 1 fimbriae exist on the surface of UPEC [23,38,43]. Furthermore, they can be up to 2 μm in length and 10 nm in width, which guarantees successful bacterial attachment. Meanwhile, numerous studies have found that over 50% of women with an acute UTI show strong positive type 1 fimbriae via the immunostaining of urine sediments [44], with results comparable to those obtained in acutely infected mice [45]. Interestingly, a study found that urine decreases the UPEC expression of type 1 fimbriae [46,47], which may explain the results above. Normally, type 1 fimbrial adhesin subunits are bound to uroplakin molecules in urothelial cells. After successful binding, the phosphorylation processes follow [48]. During phosphorylation procedures, certain signaling pathways associated with bacterial invasion, apoptosis, and the regulation of intracellular ions of calcium within urothelial cells and tissues are complete [48]. At the same time, the kidney cells secrete Tamm–Horsfall protein molecules into the urine [49]. As an 8.7 kbp operon, *fimBEAICDFGH*, which encodes type 1 fimbriae, is highly conserved. Accordingly, proteins with various functions are produced: regulatory proteins (FimB and FimE); a major subunit (FimA); minor subunits/adaptor subunits (FimI, FimF, and FimG); a chaperone (FimC); an usher (FimD); and an adhesin (FimH) (Figure 4) [50].

As a distal adhesin subunit of type 1 fimbriae, when FimH attaches to the superficial urothelial receptors, it may lead to inflammatory responses, such as dysuria and other painful symptoms in the urinary tract [36]. Numerous studies have found that the expression of FimH is required for bacterial adherence to human bladder tissue in situ [41,42], and the vaccination of mice and cynomolgus monkeys with FimH has protected them against experimental cystitis [39,40]. Interestingly, the receptor for type 1 fimbriae appears to vary depending on the state of urothelial cell differentiation. In mature superficial umbrella cells, the FimH receptor is the mannosylated uroplakin protein UPIa [32]. After binding to UPIa, the phosphorylation of UPIIIa occurs. UPIIIa is the only one of the four major uroplakins with a potential cytoplasmic signaling domain, leading to increased intracellular calcium and enhanced invasion [51,52]. However, in vitro studies found that some immature urothelial cells, such as 5637 bladder transitional carcinoma cells, generally do not express uroplakins on the cell surface, while mannosylated *α*3 and *β*1 integrins are expressed and have been shown to bind to FimH [33]. Simultaneously, bacterial invasion after FimH binding has been reported to involve components of clathrin-coated pits (for example, the cargo-adaptor protein AP-2 [53]). The adhesin subunit of FimH encompasses a lectin domain on the N-terminal head, contributing to fimbrial polymerization by attaching to the related fimbrial subunit. On the other hand, the C-terminal end is a fimbrin domain, which links to superficial glycan molecules acting as the host’s urothelial cell receptors [50]. In addition, several amino-acid residues of FimH have been found to be under positive selection [54,55,56]. Further studies have found that FimH mediates pathoadaptive mutations in UPEC clinical isolates. When mutations of these residues occurred, the virulence in a murine model of cystitis was reduced, which suggested that FimH plays an important role in human UTIs in vivo [56]. 

#### 2.1.2. P Fimbriae and PapG

The role of P fimbriae in UTIs is complex and has not yet been fully explored, but a substantial amount of evidence demonstrates its importance to UPEC [57,58]. Several studies have reported that P fimbriae are strongly related to pyelonephritis in children [14,59] but are not highly expressed in women with acute and recurrent UTIs, only accounting for 40% to 50% in isolates [60]. This is likely due to the occurrence of inflammation in acute pyelonephritis and the reduced requirement for P fimbriae in kidney colonization during severe or recurrent UTIs [50,61]. The linkage of the overwhelming majority of UPEC on urothelial cells is mediated by P fimbriae. α-D-galactopyranosyl-1,4-*β*-D-galactopyranoside, present in the globo-series glycolipids including GbO3, GbO4, and GbO5, is the receptor for urothelial cells and links with P fimbriae [43]. In humans who are non-secretors of ABO antigens, P fimbriae receptors are found more abundantly on the surface of the kidneys and lower urogenital tract urothelial cells [62]. Recent studies have revealed that many glyco(galacto)lipids exist on the surface of the kidneys’ urothelial cells, indicating the connection between the tip adhesins of P fimbriae and the superficial di-galactose molecules of glycolipid receptors in the kidneys [23]. The above connection may lead to the production of sphingolipid ceramide molecules, which activate the TLR4 signaling pathway [23]. In addition, sphingolipid ceramide molecules regulate certain immune system responses such as proinflammatory cytokines, chemokines (IL-6 and IL-8), and the activation of some groups of leukocytes [63]. Therefore, the occurrence of inflammation can contribute to the elimination of UPEC cells, but it may also harm kidney tissues, prompting the occurrence of acute pyelonephritis. This could explain why the secretor/non-secretor status of ABO antigens is a significant risk factor for UTIs. However, the chronic inflammatory changes in patients with severe or recurrent UTIs may lessen the requirement for P fimbriae when colonizing the kidney [61].

The number of *papIBAHCDJKEFG*—10 kbp operons located on pathogenicity islands (PAIs) [64]—depends on various classes of adhesin subunits (ranging from 9 to 12 genes). Accordingly, P fimbrial proteins with different functions encoded by *papIBAHCDJKEFG* are produced: a tip adhesin (PapG), adapter proteins (PapF, PapE, and PapK), a major subunit (PapA), and a terminator subunit (PapH) [50,65]. Other proteins encoded by *papIBAHCDJKEFG* include regulator molecules (PapI and PapB), an usher molecule (PapC), a chaperone molecule (PapD), and a minor subunit (PapJ) (Figure 4) [50,65]. Three alleles of the *papG* gene (PapGI, PapGII, and PapGIII) are recognized among UPEC pathotypes, which differ in their binding specificity to globosides. Among these, PapGII, the most common in UPEC strains, is identified through its receptor isotype, GbO4, and is required for the establishment of pyelonephritis and bacteremia in mammals [65,66]. PapGIII, which binds to GbO5, is involved in acute cystitis. A recent clinical study found that horizontally acquired papGII-containing pathogenicity islands underlie the emergence of invasive UPEC lineages [57]. However, in a CBA murine model of infection, a lack of PapGII did not affect pathogenesis [67]. PapGII was found to be unnecessary for robust bladder infection in a primate model of uncomplicated cystitis [2]. Alternative adhesins capable of colonizing the kidney epithelium may have influenced the above studies. Additionally, the GbO4 receptor may not be highly expressed in non-mammals [68]. Live multiphoton studies suggest that P and type 1 fimbriae may work together to colonize renal tubules by promoting bacterial attachment and biofilm growth [69]. Thus, although P fimbriae are not highly conserved and are only expressed in about half of UTI isolates, drugs targeting both type 1 and P fimbriae may provide broad bladder and kidney protection.

#### 2.1.3. Other Fimbriae and Non-Fimbrial Adhesins

The Auf fimbriae are the products of the CUP, in which periplasmic chaperones cooperate with outer-membrane usher proteins to polymerize fimbrin (pilin) subunits and produce these fimbrial organelles. More CUP-assembled pilus operons have been found in UPEC than in commensal bacteria, in addition to those expressing type 1 and P fimbriae. However, the receptors and activities of these extra UPEC-associated fimbriae have not been fully validated, and neither have those of type 1 and P fimbriae. S, F1C, and type 9 fimbriae are enriched in UPEC but less well conserved and provide a weaker contribution to biofilm formation than type 1 and P fimbriae. In frozen tissue sections, they have been shown to have the ability to bind to human kidney epithelia, and they may have different functions during different UTI phases [70,71,72]. F1C fimbriae, part of the S fimbriae superfamily, are found in about 30% of UPEC strains [29]. The adhesive virulence factor of S fimbriae has been identified in UPEC [50]. Additionally, the binding ability of S fimbriae with sialic acid molecules has led to urosepsis [38]. Type 9 fimbriae play a significant role in the formation of biofilms on abiotic surfaces, which may explain their contribution to CAUTIs [73,74]. Like Auf, F1C, S, and type 9 fimbriae, type 3 fimbriae contribute to bacterial colonization and biofilm formation processes in UPEC [50]. In addition, the Ygi pilus provided a slight advantage during kidney colonization, whereas the Yad pilus was found to contribute to the adhesion of UPEC to bladder epithelial cells in vitro but was not necessary for experimental UTI in mice [75]. While fimbriae are essential in the initial attachment of UPEC to the mucosa of the urinary tract, the bacteria also produce many outer-membrane vesicles, which may be crucial in the pathophysiology of the illness. Gram-negative bacteria produce outer-membrane vesicles at all phases of their development [76]. The creation of membrane vesicles is thought to be a “smart” technique for bacteria to protect their toxins and an effective way to transport them to the host cell [77]. TosA, a new adhesin released by a related type 1 secretion pathway, was recently reported [78]. About 30% of urinary tract isolates include TosA, which is expressed during UTIs [78,79,80]. However, the function of TosA in UTIs remains unknown. FdeC, another adhesin, is allegedly produced exclusively when in contact with host cells and is well conserved across all pathotypes of *E. coli* and intestinal commensals [81]. The presence of FdeC provided the mouse model with a competitive edge in bladder and kidney colonization, while the immunization of mice with FdeC antigens had no effect on bladder infection and only protected against kidney infection. UpaG, another autotransporter, promoted cell aggregation and the formation of biofilms and provided adherence to epithelial cells of the human bladder. However, UpaG may not be required for colonization [82]. In addition, the iron-regulated adhesin Iha was demonstrated to mediate adhesion to bladder epithelial cells and grant UPEC a significant advantage in a mouse model of a UTI [83]. An additional structure, the curli protein, is usually produced by *E. coli* and contributes to adhesion, host colonization, biofilm formation, and invasion [84,85,86]. The extracellular nucleation/precipitation mechanism is included in the pathway for curli assembly on the bacterial surface, which differs from CUP and other pilus assembly systems. Through this mechanism, curli subunit proteins are first secreted onto the cell surface, before being incorporated into the growing fiber. Several studies have demonstrated that curli recognition is mediated by TLR2, which can result in the activation of pro-inflammatory molecules such as IL-6, IL-8, and TNF-α [87,88]. Consistent with the above results, a study of the murine urinary tract reported that the curli shows multiple functions, including a pro-inflammatory response [89]. In summary, although the roles of other fimbriae and non-fimbrial adhesins have been explored, P fimbriae and type 1 fimbriae play the most important role in bacterial action.

### 2.2. Flagella-Mediated Motility

In addition to adherence, bacterial motility is another characteristic that is usually associated with the virulence of bacterial infections. Flagella are complex surface structures that mediate motility and contribute to fitness during UPEC UTIs [90,91]. The regulation of flagella and fimbriae has been shown to be coordinated. A transcriptional regulator called PapX was found to be present at the 3′ end of the P fimbrial operon in UPEC. The flagellin protein (FlaA), flagella production, and the motility of bacteria were all decreased by the overexpression of PapX [92]. In addition, as a transcriptional repressor, PapX inhibited the transcription of the *flhDC* gene, which acts as a sort of master regulator of flagellar motility [17]. Therefore, the flagella and fimbriae in *E. coli* are connected.

Furthermore, flagella-related gene expression was regulated during the growth phase of *E. coli*, indicating that flagella are involved in several functions during the processes of adhesion, maturation, and proliferation [93,94]. During the processes of adhesion and colonization, a torsional change in the outer membrane of bacteria happens after contact with a urinary catheter. This kind of change can be sensed by regulators of the flagellar master operon proteins, which induce the expression of flagella to produce the highly flagellated bacteria that are required for swarming during UTIs [1,90,91]. On the other hand, flagella participate in the formation of a multicellular bacterial community that is protected from immune responses, antimicrobial agents, and other stresses. Therefore, bacteria can mature and proliferate rapidly [1,90,91]. Additionally, the ascent of UPEC to the upper urinary system, which reaches its peak in the murine kidney, coincided with the growth of flagella [95]. The importance of flagella for kidney colonization has not been proven, though several mutants with decreased flagellar motility were not found to be consistently deficient during kidney colonization in competitive infection studies [90,96].

### 2.3. Toxins

Three main types of toxins are produced by UPEC and secreted into their surroundings: hemolysin, cytotoxic necrotizing factor 1 (CNF1), and secreted autotransporter toxins. Hemolysin, encoded by the *hlyCABD* operon, is a prototypical calcium-dependent repeats in toxin secreted protein that can penetrate uroepithelial cell membranes. Although hemolysin has not been shown to be necessary for colonization in experimental UTI, it does contribute to virulence because it is associated with renal damage and scarring. According to reports, it induces Ca^2+^ oscillations in renal tubular epithelial cells at small physiological doses, further disrupting normal urine flow, thereby enhancing UPEC ascension and colonization in the ureters and kidney parenchyma [97]. Furthermore, hemolysin was also found to induce pro-inflammatory caspase-1/caspase-4-dependent cell death in bladder epithelial cells, resulting in cell exfoliation. In addition, hemolysin can attenuate UPEC strains during acute infections in mice, suggesting that acute bladder exfoliation is a host defense mechanism [98]. CNF1 activity causes the Rho family of GTP-binding proteins to be constitutively activated, which induces host cells to remodel their cytoskeletons [99]. CNF1 has been linked to host cell invasion and cellular adhesion, which might represent fitness factors in hosts [100,101]. In addition, UPEC strains positively expressing CNF1 increase inflammation and bladder cell apoptosis through the activation of the Rho GTP-binding proteins Rac1, RhoA, and CDC42, suggesting that CNF1 might stimulate the exfoliation of bladder cells in vivo, thereby exposing underlying tissues [102]. Other UPEC toxins, i.e., serine protease autotransporters of *Enterobacteriaceae* (SPATEs) including Sat, Pic, and Tsh, have been proven to contribute to renal pathology [103,104]. Sat (secreted autotransporter toxin) mediates cytopathic effects on bladder and kidney cells in vitro and elongates kidney cells with the apparent impairment of cellular junctions [104,105]. In addition, it can degrade human coagulation factor V [106] and induce autophagic cell detachment [107]. Pic, acting as a mucinase, is expressed during infection. However, it is unnecessary for colonization [103,108]. Tsh, also called vat, (vacuolating autotransporter toxin) in CFT073, is again expressed during infection and contributes to UTIs [103,109,110].

### 2.4. Metal Acquisition

Iron, an essential nutrient, is ingested by hosts and can be acquired by bacterial pathogens. Iron acquisition is another requirement for bacterial virulence [111,112]. The most diverse and broadly distributed iron acquisition mechanisms used by bacteria are siderophore acquisition systems. The primary function of siderophores, which are small chelating molecules with a very high affinity for iron, is assumed to be scavenging ferric iron (Fe^3+^) [113]. Deleting rhyB in CFT073 reduced siderophore production and decreased bladder and kidney colonization [114]. A small regulatory noncoding RNA, rhyB, plays a role in the regulation of siderophore production in *E. coli.* Direct host iron capture occurs either from free heme or from proteins containing heme, such as hemoglobin. Heme is a direct source of iron in vivo that can be obtained via the direct heme uptake systems in UPEC. Hma and ChuA, which are iron-binding receptors, bind to heme and transport it to the periplasm. An ATP-binding cassette (ABC) transporter is then used by ChuT to mediate further transfer to the cytoplasm [115,116]. ChuA is extensively expressed in IBCs, and a deletion mutant deficient in this heme transporter greatly shrinks IBCs in vivo [114]. Additionally, microbes can sequester host iron through siderophores with high affinity, such as enterobactin, salmochelins, aerobactin, and yersiniabactin [117]. Enterobactin is broadly conserved among *E. coli* strains, whereas the production of yersiniabactin, salmochelin, and aerobactin is concentrated in UPEC. Salmochelins are encoded by the *iroBCDEN* gene cluster. By escaping the effects of the host lipocalin-2 molecule, this siderophore increases the pathogenicity of ExPEC strains [118]. Salmochelins exhibit greater affinity than enterobactin and are highly expressed at a low pH [119]. IroN, the salmochelin receptor, may have numerous functions, as it has been demonstrated to facilitate the bacterial invasion of bladder epithelial cells in vitro, and a mutant UPEC strain lacking IroN was inhibited in a cystitis mouse model [120,121]. Yersiniabactin, a known siderophore, also contributes to UPEC resistance to urine copper toxicity [122]. In addition, the harmful effects of copper (II) ions are sequestered by yersiniabactin, perhaps improving resistance to phagocyte killing [122]. Enterobactin, a highly conserved siderophore, contributes to copper sensitivity, indicating that the seeming redundancy of siderophores may be a bacterial adaptation to inhabiting different host niches.

In short, nearly all common iron-acquisition-associated genes are significantly positively selected in UPEC clinical isolates [28,123]. In the urine, bladder, and kidneys of mice, the asymptomatic bacteriuria isolate UPEC 83972 outcompeted a mutant strain lacking salmochelin and enterobactin [124]. Aerobactin appears to be crucial for bladder fitness in the pyelonephritis isolate CFT073, which does not manufacture yersiniabactin, indicating that these two siderophores may have overlapping roles [125]. As a result, multiple bacterial iron acquisition mechanisms have been chosen for UPEC, perhaps in part because of their function in IBC formation, which may make targeting them with vaccines or therapeutics difficult. This problem might be solved by including multiple siderophore receptor antigens in a single vaccination. However, the TonB inner-membrane protein is necessary for all siderophore receptors to transmit the energy required for import. TonB deletion from a UPEC strain was reported to significantly decrease pathogenicity in the kidney and, to a lesser extent, the bladder [126]. Therefore, discovering small molecules or medicinal plants to target TonB inhibition may be a successful anti-infection strategy.

### 2.5. Intracellular Bacterial Communities (IBCs)

**Figure 5:**
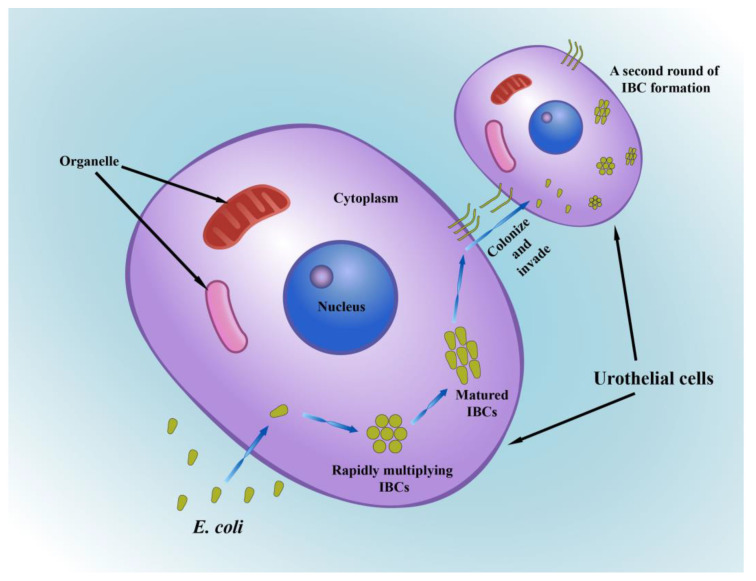
The formation of intracellular bacterial communities (IBCs). *E. coli* enter the cytoplasm of urothelial cells and then form IBCs. The rapidly multiplying *E. coli* initially adopt a coccoid morphology and then change back to a rod-like shape as the IBC mature. Finally, *E. coli* protrude from the original cells and colonize and invade nearby cells in the form of filaments, initiating a second round of IBC formation.

*E. coli* can enter the cytoplasm after adhering to and being taken up by the host cell, where they multiply rapidly. The bacteria eventually multiply excessively over several generations and form IBCs, which are distinctive in the acute stage of infection [127]. Additionally, dispersal from the IBC is essential for bacterial persistence. During the first 12–16 h of an experimental mouse bladder infection, an only partially understood differentiation program contributed to IBC maturation. The rapidly multiplying bacteria initially adopted a coccoid morphology, then changed back to a rod-like shape as the IBC matured, and finally fluxed away from the IBC. Then, frequently in the form of filaments, UPEC protruded from the dying urothelial cells and colonized and invaded nearby cells, initiating a second round of IBC formation (Figure 5) [127]. Experimental UPEC infection in mice did not fully recapitulate IBC development. In urine sediments from women with recurrent cystitis, IBCs have been identified through translational investigations, while evidence of IBCs was not found when the UTI pathogen was a Gram-positive organism [128]. In addition, the IBC pathway is also used by other Gram-negative uropathogens expressing type 1 fimbriae, including *Klebsiella pneumonia*, *Enterobacter* spp., and *Citrobacter freundii* [96]. These findings collectively imply that the IBC pathway is a key mechanism for the establishment of UTIs in mammalian bladders by Gram-negative uropathogens expressing type 1 fimbriae. Additionally, IBCs with biofilm-like structures are shielded from components of the host immune response, including neutrophils and antibiotics [129]. Less differentiated cells in the lower levels of the urothelium become exposed to bacteria at the luminal surface because of the loss of superficial cells during the exfoliation process. As a result of the denser actin network in these cells, UPEC cells are not able to replicate at these sites [130]. These aggregates of quiescent UPEC cells have been called quiescent intracellular reservoirs (QIRs) [131]. The emergence of either IBCs or QIRs contributes to the recurrence of UTIs, owing to bacteria fluxing out of infected host cells and invading new cells [127]. In conclusion, the IBC pathway is a significant target for therapeutic intervention.

### 2.6. Strategies for Evading Host Defenses

The infection causes the host to have a significant pro-inflammatory reaction, which is followed by an inflow of neutrophils and attempted eradication of bacteria. Unlike commensal strains, UPEC may be able to block this induction of pro-inflammatory mediators in addition to colonizing the host and causing damage to the nearby tissue. Another strategy is escaping from immune recognition by either hiding intracellularly or masking the immunogenic surface structures.

Some fitness and virulence factors may have characteristics that shield UPEC from the host immune response. For instance, as mentioned above, iron is a crucial nutrient that UPEC must collect during infection, and the siderophore enterobactin is one route of iron acquisition. However, the neutrophil-expressed and neutrophil-released host protein lipocalin-2 specifically binds to and sequesters enterobactin, preventing it from providing UPEC with iron [132]. Enterobactin can be modified by UPEC through glycosylation, and this modified enterobactin, called salmochelin, is not bound by the host protein lipocalin-2 and thus is not inhibited in its iron-sequestering function [133,134].

The major component of the cell wall in Gram-negative bacteria is LPS (also called endotoxin), which is highly immunogenic. The hydrophobic lipid A, core oligosaccharide, and repeats of O-antigen subunits make up the structure of LPS [135]. The highly conserved outer leaflet of the membrane contains the hydrophobic lipid A, which mediates the toxicity of LPS. The O antigen, which is exposed at the surface of the bacteria and constitutes the major immunogen, is bound to the oligosaccharide core [136]. Thanks to several LPS O antigens, UPEC has the capacity to dampen the induction of cytokines and chemokines in epithelial cells [137,138]. The ability of UPEC strains to inhibit the induction of interleukin-6 (IL-6) and IL-8 is neutralized by the disruption of the *rfa* operon, particularly the deletion of *waaL*, which codes for the O-antigen ligase [137,138]. In vivo, this process is accompanied by increased neutrophil recruitment and elimination [137]. Additionally, serum resistance is linked to a few O antigens that are often detected in UPEC [139]. For instance, the cystitis strains UTI89 and NU14 belong to the O18 serotype, while the pyelonephritis strains CFT073 and 536 belong to the O6 serotype. The O18 and O6 serotypes are both associated with serum resistance [139].

Biofilm and extracellular matrix components have also been implicated as contributors to evading host defenses. In biofilms, bacterial communities are formed and embedded in an extracellular matrix, which is mainly composed of exopolysaccharides, nucleic acids, and proteins. Bacteria within biofilms are effectively sheltered against adverse environmental conditions, including antimicrobial treatment and endogenous host immune defense [140]. Therefore, the ability of bacteria to form a biofilm could be attributed to the combined action of adhesion, the production of an extracellular matrix, and growth characteristics. The pathogenesis of several diseases, including chronic mastitis and pyelonephritis, is associated with the formation of biofilms by bacteria [141]. Additionally, UPEC produce more biofilms in vitro than commensal fecal isolates, proving that biofilm contributes to *E. coli* urovirulence [89]. Moreover, the increasing production of exopolysaccharides and biofilms in UPEC can result in more severe and persistent infections [142,143,144,145]. It is surprising that persisting UPEC isolates do not consistently produce more biofilms in vitro than isolates from sporadic infections, suggesting that in vitro biofilm formation does not necessarily reflect the in vivo situation [145]. Generally, exopolysaccharides can promote the long-term colonization and persistence of UPEC in the urinary tract. Among them, the most noteworthy exopolysaccharides are capsular polysaccharides [146], poly-*N*-acetyl glucosamine [147], and cellulose [89]. By covering highly immunogenic structures, cellulose can reduce the immune response to bacteria, and other exopolysaccharides may have similar functions.

Interestingly, the phase variation of type 1 fimbrial genes may help UPEC evade immune system recognition [17]. A promoter on an invertible element upstream of the target genes in UPEC regulates the expression of type 1 fimbrial operons, and the orientation of the promoter is controlled by multiple recombinase enzymes. Two of these enzymes, FimB and FimE, are encoded upstream of the fimbrial operon [148]. In addition, IpuA and IpbA, two additional recombinases, mediate the switching of the invertible element independent of FimB and FimE [149]. In contrast to FimE and IpbA, which can only turn expression off and on, respectively, FimB and IpuA can mediate the bidirectional switching of the promoter. This process is regulated in a complex manner and is influenced by a multitude of environmental factors, such as temperature, osmolarity, pH, and oxygen levels. Furthermore, at least three regulatory proteins are also involved, including Lrp, IHF, and H-NS [17]. When the promoters are in the ‘on’ position, the genes expressing fimbriae are transcribed, and the bacteria can be recognized by antibodies. On the contrary, bacteria with their promoters in the ‘off’ position are not recognized by these antibodies, thus essentially evading the host defense.

## 3. Treatment of Urinary Tract Infections

Antibiotics have been used to combat UTIs since the introduction of sulfonamides in the 1940s. Until now, antibiotics have remained the most recommended therapeutic for UTIs [150]. However, increasing antibiotic resistance and high recurrence rates have significantly impacted the social burden of UTIs. In addition, the excessive use of antibiotics can cause long-term changes in the normal microbiota of the vagina and gastrointestinal tract and lead to liver and kidney damage, a flora imbalance, and other problems. Ideally, alternative therapies will be developed to combat the development of resistance and increase the effectiveness of antibiotics. Many promising approaches are under development, ranging from leveraging UTI pathogenesis to targeting virulence pathways. In theory, these antimicrobial therapies should be effective in reducing the ability of UTI pathogens to cause disease without causing other adverse effects. Furthermore, alternative therapies should target specific processes critical to UTI pathogenesis.

Below, we summarize the status of the use of antibiotics as traditional pharmacologic therapies and discuss the current challenges that have arisen from the emergence of multidrug-resistant bacterial strains. We emphasize the significant potential of vaccines and medicinal plants with antibacterial activity as anti-UTI medicines.

### 3.1. Antibiotics and Multidrug Resistance

Currently, patients with clinically symptomatic UTIs are usually treated with antibiotics. Several factors should be considered in the selection of the appropriate therapy, including host factors (such as sex, the potential for adverse effects, a compromised immune system, or urologic abnormalities), the severity of the illness (such as the duration of therapy), and the risk of multidrug resistance (such as the activity spectrum of the antimicrobial agent and resistance prevalence for the community). In addition, the management of UTIs is influenced by the increasing prevalence of resistant organisms and the potential for the spread of resistance among normal host flora with the use of broad-spectrum antibiotics [151,152].

Four agents are recommended for first-line therapy: nitrofurantoin, trimethoprim-sulfamethoxazole (TMP-SMX), pivmecillinam, and fosfomycin tromethamine. Two alternative agents are also recommended: *β*-lactams and fluoroquinolones (Table 1) [151,152,153]. An important recommendation should be noted. Owing to the risk of serious harm outweighing the benefits, fluoroquinolones (for example, ciprofloxacin) have been transferred to the latter category of agents and should only be used when no other effective oral options are available. Nitrofurantoin has been an option for the management of UTIs for more than 70 years. However, nitrofurantoin does not achieve high serum concentrations and has poor tissue penetration. Therefore, it should be avoided if pyelonephritis is possible and is contraindicated in patients with renal failure [153]. The adverse effect profile of nitrofurantoin includes nausea, headache, and gastrointestinal effects. Higher doses or longer durations of use may lead to significant hepatic and pulmonary toxicity [153]. Nitrofurantoin affords comparable efficacy to TMP-SMX for UTIs in terms of both clinical and microbiological cures [154,155]. TMP, with or without SMX, has been the mainstay therapy for UTIs for the past 40 years. It is also the first-line drug for acute UTIs and pyelonephritis. However, the main limitation of using TMP–SMX is the increasing rate of resistance among uropathogens [156]. The efficacy of pivmecillinam for the empirical treatment of acute uncomplicated UTIs was initially reported in clinical trials published in the 1970s [157]. Several studies have confirmed earlier reports and clinical experience suggesting that pivmecillinam is effective and well tolerated for the treatment of acute cystitis in women [157]. Furthermore, the resistance rate of pivmecillinam is lower than that of other recommended agents and is relatively stable over time [158]. Fosfomycin tromethamine, a soluble salt of fosfomycin, is approved for the treatment of uncomplicated UTIs in women. Although surveys show that it remains active against other antibiotic-resistant Gram-negative organisms, data supporting its efficacy for the treatment of MDR-uropathogens are limited [159,160]. Given the concerns that its widespread use will increase drug resistance, it is not a popular first-line choice in the treatment of complicated UTIs [161]. *β*-lactams and fluoroquinolones are second-line therapies. *β*-lactams show increased adverse effects compared with other choices, while the risk of fluoroquinolones may outweigh their benefits for treatment [151,161].

**Figure 6:**
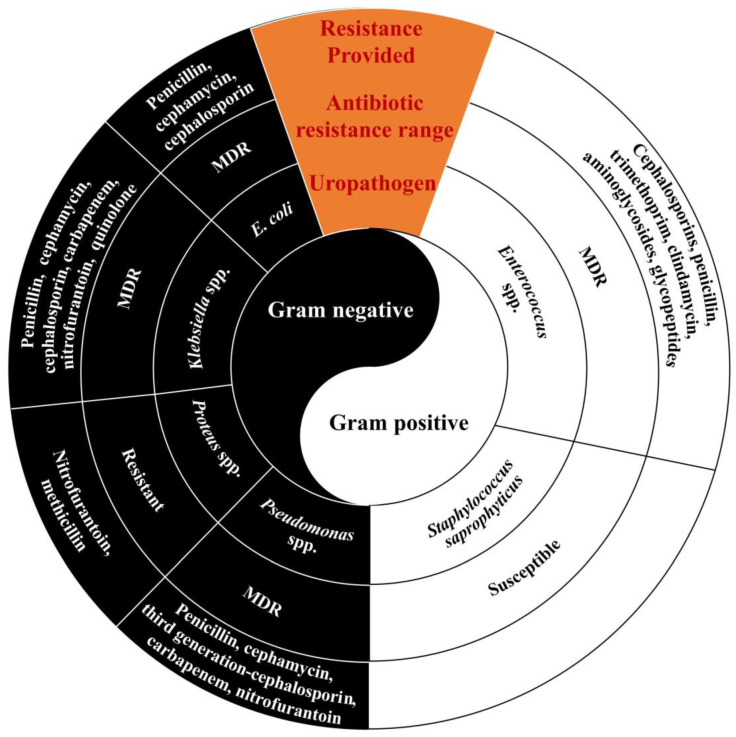
Resistance profiles for uropathogens.

**Figure 7:**
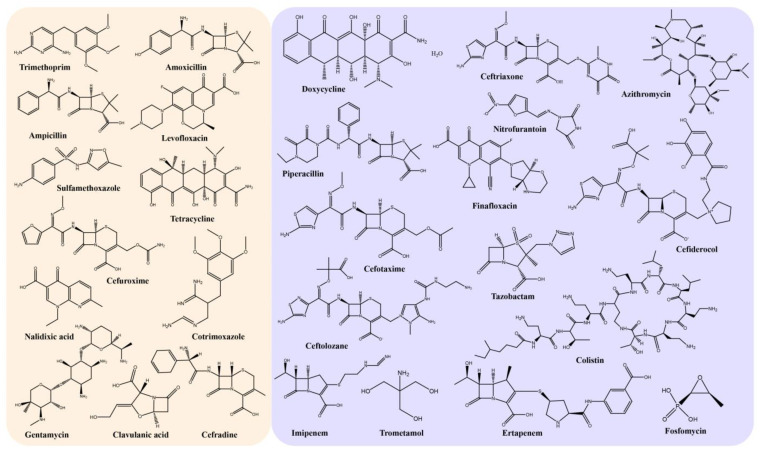
Structural formulae of some UPEC-resistant and UPEC-susceptible antibiotics (orange: UPEC resistant; purple: UPEC susceptible).

However, owing to the widespread emergence of antibiotic-resistance mechanisms (Figure 6), the treatment of UTIs is becoming increasingly difficult [3,150,162,163,164,165,166,167]. Currently, UPEC isolated from clinics contain various types of plasmids encoding extended-spectrum *β*-lactamases (ESBLs), which include temoniera (TEM), sulfhydryl variables (SHVs), oxacillinases (OXAs), cefotaximases (CTX-Ms), AmpC-type *β*-lactamases, and carbapenemases. Originating in *Klebsiella pneumoniae* and *Escherichia coli*, ESBLs are now widespread across the family of Enterobacteriaceae. ESBLs are *β*-lactamases with broad activity against penicillins and cephalosporins. They are encoded by plasmids or chromosomes and function by splitting the amide bond of the *β*-lactam ring, thus inactivating *β*-lactam antibiotics [166]. Among them, TEM and SHVs are common in Asia [168], and CTX-Ms are the most prevalent *β*-lactamases in community-associated isolates [166]. OXAs are typically encoded by plasmids, which can hydrolyze *β*-lactam rings, thereby increasing the resistance of ampicillin, cephalothin, and oxacillin [163]. AmpC enzymes, typically encoded by chromosomes, hydrolyze almost all third-generation and extended-spectrum cephalosporins and are resistant to *β*-lactamase inhibitors [166]. The carbapenemase ESBLs confer resistance to carbapenems and extended-spectrum cephalosporins [166]. These plasmids have rapidly spread resistance to third-generation cephalosporins and other antibiotics [162,164,165,167,169]. Several studies have reported that UPEC isolates are resistant to ampicillin [166], oral first-generation cephalosporins, TMP-SMX [156], cefuroxime [170], cotrimoxazole [171], amoxicillin-clavulanate [169], nalidixic acid [172], cefradine [173], and aminopenicillins [174]. Figure 7 depicts the representative antibiotic structural formulations whose resistance to UPEC has been demonstrated (orange background) and those showing susceptibility to UPEC (purple background). All in all, the troubling trend toward a high prevalence of multidrug-resistant uropathogens has stimulated the development of alternative control measures and treatment options. 

In short, with the widespread use of antimicrobial drugs and the increasing number of drug-resistant strains, the “golden age” of antibiotics is waning, and fewer antibiotics are available. To minimize the above problems, antibiotic selection should be based on local sensitivity patterns and adjusted once culture results are available. Furthermore, the unnecessary treatment of asymptomatic bacteriuria should be avoided. In addition, the disturbing trend toward a high prevalence of multidrug-resistant uropathogens has stimulated the development of complementary and alternative medicines. 

### 3.2. Preventative Strategies and Alternative Therapeutics for Urinary Tract Infections

Antibiotics will continue to be an unavoidable resource for the treatment of UTIs on a case-by-case basis. However, numerous effective strategies have been developed by bacteria to overcome therapeutic procedures and survive in the urinary system [175]. Moreover, excessive use of antibiotics can lead to the occurrence of drug resistance and interfere with the intestinal microbiota [176]. Therefore, it is necessary to search for preventative strategies and alternative remedies. In recent years, a plethora of potential drugs have been found to inhibit bacterial adhesion or weaken the effect of siderophores—such as vitamin D, estrogens, and probiotics. However, these drugs are still in the preclinical research stage as a means of preventing or treating UTIs. Therefore, the advantages of vaccines and medicinal plants are being considered. Medicinal plants, used in many countries as an alternative treatment for UTIs, contain a wide variety of phytochemicals and secondary metabolites, which are the most promising sources of compounds exerting antibacterial activity. Here, we summarize the most effective alternative remedies against UPEC, focusing on vaccines and medicinal plants.

#### 3.2.1. Vaccines

The creation of vaccines to prevent UPEC UTIs is of great clinical and academic importance due to the serious consequences of these infections and the current dearth of efficient, nonantibiotic prophylactic measures. These immunizations will likely help certain patient populations. UPEC vaccination would undoubtedly be a major benefit for patients, particularly those who suffer from recurrent infections or are more likely to develop infections. The following elements should be considered when developing an effective UPEC vaccine strategy: (a) the heterogeneity of UPEC strains; (b) the production of multiple virulence factors by UPEC strains; (c) potential adverse effects on the commensal microbiota of the intestine; and (d) comprehensive and efficient drug effects for both the upper and lower urinary tract [102,177]. An efficient vaccine should be able to generate a protective immune response against important virulence factors during particular phases of UTI pathogenesis, such as colonization, invasion, and the formation of IBCs [177]. Among the steps involved in UPEC infections, adhesins, antimicrobial peptides (AMPs), and siderophores are the most likely candidates for target antigens [20].

In general, vaccines to prevent UPEC can be divided into two categories: whole-cell vaccines and antigen-specific vaccines (Table 2). The most effective vaccinations created to date are whole-cell vaccines. Whether attenuated or inactivated, vaccines containing intact uropathogens expose the host to a variety of virulence factors that trigger the body’s immune response, thus achieving the goal of vaccination. Solco-Urovac, a polyvalent inactivated vaccine, was developed for humans after several decades of research in animals. It consists of ten distinct strains of heat-inactivated bacteria: six from UPEC and one strain each from *Enterococcus faecalis*, *Proteus mirabilis*, *Morganella morganii*, and *Klebsiella pneumoniae*. The vaccine was reported to protect Europeans from recurrent UTIs in a clinical trial [178]. However, it produced significant adverse effects, such as nausea, fever, burning, bleeding, and vaginal itching [179], which might have led to its abandonment. StroVac, currently available for use in humans, is another intramuscular polyvalent inactivated UTI vaccine that comprises the same ten UPEC strains in a different formulation and produces few side effects in the nonantibiotic prophylaxis of recurrent UTIs [180,181]. Another oral inactivated UTI vaccine, Uro-vaxom, comprises membrane proteins of 18 UPEC strains. It can protect women from recurrent UTIs, has few side effects, and has been approved for use in Europe since 1994 [182,183,184]. However, its major drawback is poor patient compliance because it requires repeated injections every three months, which limits its promotion [185]. Other inactivated vaccines against UTIs include Urvakol, Urostim [186,187], ExPEC9V [188], and CP923 [189], which promote immunogenicity in animals but have not completed clinical trials. NU14 ∆*waal*, the representative attenuated vaccine for UPEC, is a whole-cell vaccine. It was identified from a mutant of the UPEC strain NU14 that lacks the O-antigen ligase *waal*. In a murine UTI model, NU14 ∆*waal* was considerably less virulent and more inflammatory than wild-type NU14. When challenged with infection by NU14, CFT073, and four other UPEC isolates, mice were protected from infection by vaccination with NU14 ∆*waal* [190]. However, the potential of NU14 ∆*waal* was diminished by the discovery that serial infections with NU14 ∆*waal* cause chronic bladder pain [191].

In addition, antigen-specific vaccines have been investigated as potential prevention targets, including capsular- or LPS-based, fimbrial, and non-fimbrial adhesin, iron-scavenger-receptor-based, and toxin-based vaccines [2,205]. ExPEC4V, a polysaccharide-based vaccine containing the O antigens of four *E. coli* serotypes (O1A, O2, O6A, and O25B), induced significant IgG responses and significantly reduced the incidence of UTIs caused by UPEC even with higher bacterial doses compared with the placebo group in phase II clinical trial [196,197,205]. Adhesins that promote the UPEC colonization of the bladder epithelium also cause host immune reactions. Therefore, adhesins can be used to create a desirable antigen candidate that can prevent adhesin-host cell-receptor interaction and obstruct the bacterial colonization of the host [50]. In animal models, the FimH adhesin from type 1 fimbriae played a crucial role in the pathogenesis of UPEC in the lower urinary tract. It has proven to be highly efficient in treating mice when challenged with bacteria cultured under conditions that induce type 1 fimbriae [206]. FimH can be purified either in its mannose-binding native form or attached to its periplasmic chaperone FimC (FimCH) [44]. In a mouse model, both antigens were effective at preventing the colonization of various UPEC strains [206]. Numerous preclinical investigations have demonstrated the effectiveness of FimH vaccinations in triggering an immune response and preventing UPEC. Both approaches were reported to be effective at preventing cystitis when a recombinant FimH vaccine was administered to mice either intranasally or intramuscularly [207]. Another report showed that the subcutaneous administration of recombinant FimH coupled to the flagellin subunit FliC, a TLR5 agonist and potential adjuvant, shielded mice from cystitis when challenged with a type 1 pilus-expressing clinical strain [198]. Additionally, in murine and primate models, a FimH vaccination with alum and MF59 adjuvants or with FliC and Montanide ISA 206 adjuvants induced an immune response to UPEC and inhibited colonization [199,208]. Last, a team created a FimH plasmid construct with mammalian codon optimization for use in a DNA vaccine, allowing for the induction of a protective immune response in mice when plasmid DNA was injected [209,210]. Mice that received the DNA vaccination through footpad injection presented much less extensive bladder colonization and significantly higher urine IgA titers [211]. However, most of these vaccines were later rejected due to the lack of efficacy in humans after FimH vaccination tests in clinical trials. The failure of the FimH-based vaccination was due to several factors. UPEC strains are not consistently detected by the immune system due to the variable expression of type 1 fimbriae in animal and human models [200]. In addition, antigens developed against the fimbriae do not target their mannose-binding region. Multiple studies utilizing a range of target antigens in animal urinary tract models may be necessary to develop an efficient UTI vaccine [2,200]. A TLR ligand-based vaccine using FimH adhesin is currently being explored, with the fusion of FimH adhesin to the flagellin of UPEC as a TLR5 ligand able to elicit an immune response and shield mice from UTIs [198,201,202]. A study reported that cynomolgus monkeys receiving the FimCH vaccination intramuscularly were shielded from pyuria and bacteriuria [39]. In addition, in mouse and primate models, the FimCH vaccine with Freund’s adjuvant induced an immune response against UPEC and inhibited colonization [199,208]. However, the development of the FimCH vaccine was abandoned during phase II trials despite its safety being confirmed in phase I studies. Evidently, whether a vaccine is composed of truncated FimH or a complex of FimH with the chaperone FimC, it will eventually be rejected due to a lack of efficacy or adverse reactions in clinical trials. Moreover, the PapG adhesin from P fimbriae also plays an important role in UPEC pathogenesis. Analogous to the FimH and FimCH vaccines, a vaccine composed of truncated PapG or a complex of PapG with the chaperone PapD (PapDG) was able to protect cynomolgus monkeys from pyelonephritis [212]. In addition, PapDG vaccines with Freund’s adjuvants were also shown to generate immune responses and reduce UPEC colonization in a mouse model [20].

The use of siderophore–protein conjugates was found to induce immune responses targeted at bacterial siderophores and to successfully protect against UTIs [213], meaning that *E. coli* iron acquisition systems have the same potential as UPEC vaccine candidates. IroN, IutA, IreA, and FyuA increased protection in the urinary tracts of mice in a study using an attenuated Salmonella vaccine delivery system to assess the efficacy of a multi-epitope vaccine comprising siderophore receptors [214,215]. In a mouse model, researchers discovered that systemic vaccination with the salmochelin receptor IroN or IroN with Freund’s adjuvant could offer protection against UTIs [121], while the aerobactin receptor IutA conjugated with cholera toxin also produced potent immune responses and provided defense against UPEC infection [203]. The intranasal administration of the yersiniabactin receptor FyuA prevented mice from contracting pyelonephritis after exposure to UPEC strain 536, which expresses FyuA. FyuA also produced potent immune responses and provided defense against UPEC infection in mouse models when used with alum as an adjuvant [204]. Additionally, the potential antigen Hma, a heme receptor, exhibited kidney protection against challenge infection [216]. In a similar vein, UTI symptoms were reduced in murine experimental infection models by a toxin-based vaccine including antigens such as hemolysin HlyA, recombinant hemolysin, and mutant CNF1 and HlyA toxins [2,200]. As potential vaccine targets, autotransporter toxins such as FdeC from UPEC strains have also been studied. Identifying whether these results extend to human UTI patients should undoubtedly be a future study objective.

#### 3.2.2. Medicinal Plants for the Management and Treatment of Urinary Tract Infections

With the increasing prevalence of UTIs and the economic burden for both patients and governments, developing effective anti-UTI drugs that can be quickly applied in clinical settings is important. Vaccines and small molecule formulations may be a promising option; however, the high cost (over one billion USD) and lengthy process of developing new antibacterial agents (more than 10 years) must be taken into consideration [217]. Therefore, medicinal plants have attracted increasing interest within the scientific community. In fact, research on medicinal plants has grown significantly since the beginning of the 21st century, touching on their effectiveness in treating and preventing UTIs, their therapeutic material basis, and their mechanisms of action [218].

The superiority of medicinal plants has also attracted increasing research interest. As plant extracts contain a variety of phytochemicals that act against different molecular targets within bacterial cells, bacteria are less likely to develop resistance to medicinal plants, and thus they can maintain increased efficacy and decreased side effects [219,220,221]. Medicinal plants contain a wide variety of phytochemicals and secondary metabolites and are classified according to their chemical composition, including alkaloids, coumarins, flavonoids, lectins, polypeptides, quinones, tannins, and terpenoids [222]. The parts of the plant used to treat UTIs vary substantially, as do the chemical compositions of these parts. In addition, the plant extraction and processing methods also impact the chemical composition. Generally, the plant part, form, and dosage depend on information gathered from folk medicine or the experience of ethnophysicians. Therefore, fully developing and applying medicinal plants is a significant challenge.

Although the exact mechanisms by which plants offer curative or preventative effects against UTIs are unclear, substantial research is ongoing in this field. Studies have shown that many medicinal plants exert antibacterial activity against a variety of bacterial pathogens, and combined use with antibiotics can enhance bactericidal and bacteriostatic effects [223]. In some cases, medicinal plants can even reverse antibiotic resistance. In addition to bactericidal and bacteriostatic effects, medicinal plants have been reported to present other mechanisms, such as antiadhesive activity [224,225,226], the inhibition of bacterial growth [227,228], anti-biofilm-formation activity [229,230], the inhibition of motility [231,232], the protection of host urinary cells [233], and the enhancement of antibacterial immunity. In Table 3, we summarize recently studied plants that achieved positive results against UPEC. We hope that this will help in the selection of clinical anti-UPEC drugs.

## 4. Conclusion and Future Prospects

In this review, we described an interaction network comprising UPEC, the host, and antibiotics. Vaccines and medicinal plants were also included as preventative strategies and alternative therapeutic methods for UPEC. The main pathogen of UTIs is UPEC, which can survive within the urinary tract and cause infection through a wide range of virulence factors, including fimbrial and non-fimbrial adhesins, flagella, toxins, lipopolysaccharides, surface vesicles, polysaccharide capsules, and the iron acquisition system. Hence, as vital organs, the urethra, bladder, and kidneys are often attacked by UPEC. The pathogenesis of a UTI starts with the contamination and colonization of the urethral area with UPEC. The bladder epithelium is then invaded by UPEC. Finally, UPEC release toxins that damage host tissues, resulting in upper UTIs. Severe cases can also result in life-threatening illnesses, such as bacteremia, septicemia, and urosepsis. Since the 1940s, when sulfonamides were first introduced, various antibiotics have been employed to treat UTIs and then UPEC. Until now, antibiotics have remained the most frequently recommended treatment for UTIs. However, as new and more severe diseases have emerged, the growing resistance to antibiotics has led to the recurrence and chronicity of infection, making treatment challenging and complicated.

Therefore, the highest priority in UTI research should be the development of new, effective alternative therapies, as these therapies have the potential to significantly improve the quality of life for millions of humans and reduce the overall use of antibiotics. However, the promotion of vaccines faces numerous obstacles. The notion that any novel vaccine or therapy by itself can entirely eradicate recurrent UTIs in all patients is doubtful due to the substantial contribution of the host to recurrent UTIs unless the vaccine/therapy is also able to alter the innate mucosal-immune response to uropathogens. In the urogenital niche of hosts, UPEC can predominate in the absence of immunization, but after vaccination, other uropathogens may take the position of UPEC. Furthermore, the process of developing new vaccines for the market is relatively lengthy and expensive.

Thus, a new, more efficacious, and cost-effective strategy should be developed to combat UTIs caused by UPEC. Some of the most promising sources of compounds that exert antibacterial activity against UPEC are medicinal plants. Plants have been used extensively to treat and prevent diseases for a long time. The development of reliable and efficient plant-based medicinal treatments would undoubtedly necessitate a complete understanding of the mechanisms through which medicinal plants affect UPEC and the human body. An overview of the antibacterial activities of medicinal plants that work against UPEC was provided in this review. However, to completely understand how these plants prevent or treat UTIs caused by UPEC, more research is required.

## Figures and Tables

**Table 1:** Oral treatment regimens for acute uncomplicated cystitis.

Drug		Dose and Duration	Common Adverse Effects
Nitrofurantoin monohydrate macrocrystals		100 mg twice daily for 5 d	Nausea, headache, gastrointestinal effects
Pivmecillinam		400 mg twice daily for 5 d	Rash and gastrointestinal upset, including nausea and vomiting
Trimethoprim- sulfamethoxazole		160/800 mg twice daily for 3 d	Rash, urticaria, nausea, vomiting, hematologic signs
Fosfomycin tromethamine		3 g single-dose sachet	Diarrhea, nausea, headache
*β*-Lactams		The dose varies by agent from 5 to 7 d	Diarrhea, nausea, vomiting, rash, urticaria
Fluoroquinolones	Ciprofloxacin	500 mg twice daily or 1000 mg once daily for 3 d	Nausea, vomiting, diarrhea, headache, drowsiness, insomnia, tendon rupture, neuropathy
	Norfloxacin	400 mg twice daily for 3–7 d	
	Ofloxacin	200–400 mg twice daily	
	Levofloxacin	750 mg once daily for 3 d	

**Table 2:** Reports describing vaccines against *E. coli*, especially UPEC.

Type of Vaccine		Component of Vaccine	Comments	References
Whole-cell vaccines	Inactivated vaccines	Solco-Urovac: 10 strains of heat-inactivated uropathogens (6 from *E. coli* of different serotypes and 1 each from *K. pneumoniae*, *P. mirabilis*, *M. morganii*, and *E. faecalis*).	Causes the prophylaxis of recurrent UTIs and major side effects, such as fever, bleeding, burning, nausea, and vaginal itching.	[179,192,193]
		StroVac: the same 10 strains in a different formulation.	Causes the prophylaxis of recurrent UTIs.	[181]
		Uro-vaxom: 18 UPEC strains.	Prevents recurrent UTIs in women.	[182,183,184]
		Urvakol: a mixture of inactivated bacterial pathogens including *E. coli*, *P. mirabilis*, *E. faecalis*, *K. pneumoniae*, and *P. aeruginosa*.	Promotes immunogenicity in animals and humans.	[186,187]
		Urostim: a mixture of inactivated bacterial pathogens including *E. coli*, *P. mirabilis*, *E. faecalis*, and *K. pneumoniae*.	Promotes immunogenicity in animals and humans.	[186,187]
		ExPEC9V (NCT04899336).	Targets invasive extraintestinal pathogenic *Escherichia coli* disease (IED).	[188]
		Intranasal vaccination with formalin-inactivated CP923.	Shows stronger systemic antibody response.	[194]
	Attenuated vaccines	NU14 Δ*waaL*: a mutation of O antigens of UPEC strains.	Protects against NU14, CFT073, and four UPEC isolates.	[195]
Antigen-specific vaccines	Capsular- or LPS-based vaccines	ExPEC4V (NCT03500679): O antigens specific to serogroups O1A, O2, O6A, and O25B.	Prevents UTIs even with high bacterial doses and low bacteremia.	[196,197]
	Fimbrial and non-fimbrial adhesin vaccines	FimCH vaccine with Freund’s adjuvants; FimH vaccine with Alum and MF59 adjuvants.	Promotes an immune response against UPEC and prevents colonization in murine and primate models; ineffective in humans.	[198,199,200]
		TLR ligand-based vaccine with the fusion of FimH adhesin; vaccination with admixed FimH, FliC, and Montanide ISA 206 adjuvants.	Induces an immune response and protects mice against UTIs.	[198,201,202]
		PapDG vaccines with Freund’s adjuvants.	Generates immune responses and reduces UPEC colonization in a mouse model.	[20]
	Iron-scavenger-receptor-based vaccines	Salmochelin receptor IroN or IroN with Freund’s adjuvant; aerobactin receptor IutA conjugated with cholera toxin; yersiniabactin receptor FyuA with alum as an adjuvant.	Generates strong immune responses and protection against UPEC infection in murine models.	[121,200,203,204]
	Toxin-based vaccines	Vaccine containing antigens such as hemolysin HlyA, recombinant hemolysin, mutated CNF1, and HlyA toxins.	Reduces UTI symptoms in murine experimental infection models.	[2,200]

**Table 3:** Reports describing medicinal plants against *E. coli*, especially UPEC.

No.	Botanical Name	Part Used	Main Compound Class	Effect and Mechanism	Proof Obtained	References
1	*Abelmoschus manihot* (L.) Medik.	Flowers	Phenolics	Acts against LPS-induced cystitis; attributed to its anti-inflammatory profile by suppressing TLR4/MYD88/NF-*κ*B pathways.	In vivo	[234]
2	*Agropyron repens* (L.) P. Beauv.	Rhizome	Phenolics, flavonoids	Decreased bacterial adhesion; interaction with bacterial outer-membrane proteins.	In vivo	[235,236]
3	*Alchornea cordifolia* (Schumach. and Thonn.) Müll.Arg.	Leaves, stem bark	Terpenoids, phenolics	Antibacterial activity on ESBL-producing *E. coli* isolates.	In vitro	[237]
4	*Andrographis paniculate* (Burm. F.) Nees	Leaves	Terpenoid	Inhibition of LPS-induced iNOS and COX-2 protein expression; negative regulation involving STAT3 phosphorylation and NF-κB activation.	In vitro	[238]
5	*Arctostaphylos uva-ursi* (L.) Spreng.	Leaves	Phenolics	UTI control; shrinking and tightening of mucous membranes.	In vitro	[239,240]
6	*Aristolochia indica* L.	Whole plant	Aristolochic acid analogs	Antibacterial activity against MDR UPEC.	In vitro	[241,242]
7	*Armoracia rusticana* (Lam.) P. Gaertner et Schreb.	Roots	Isothiocyanates	Possible damage to the cell membrane.	In vitro, in vivo, clinical	[243,244]
8	*Arnica montana* L.	Flowers	Terpenoids	Biofilm-modulating activity on UPEC.	In vitro	[229,230]
9	*Avicennia marina* (Forsk.) Vierh.	Leaves	Phenolics	Antibacterial activity.	In vitro	[245]
10	*Betula pendula* Roth.	Leaves	Phenolics	Bactericidal activity; modifications to the bacterial surface structures responsible for binding to the occupied surface.	In vitro	[246]
11	*Boerhaavia diffusa* L.	Hairy root, root	Phenolics	Active against UPEC MDR strains.	In vitro	[247,248]
12	*Bridelia ferruginea* Benth.	Leaves	Flavonoids, phenolics	Antibacterial activity.	In vitro	[249,250]
13	*Calluna vulgaris* Salisb.	Leaves, flowers	Phenolics	Antibacterial activity.	In vitro	[251]
14	*Citrus reticulata* Blanco	Seeds	Flavonoids, volatile oils	Reduction in UPEC invasion; decreased β1 integrin expression.	In vitro	[252,253]
15	*Costus spicatus* (Jacq.) Sw.	Leaves	Phenolics	Antimicrobial activity; correlation between the antioxidant and antimicrobial activity.	In vitro	[254]
16	*Crateava nurvala* Buch-Hum (Varuna)	Bark	Alkaloids, saponins	Growth inhibition.	In vitro, clinical	[255,256]
17	*Curcuma longa* L.	Rhizome	Phenolics	Antibiofilm activity; the inhibition of swimming and swarming behavior; the enhanced susceptibility of UPEC to antibiotics; alterations to biofilm morphology, including a reduction in thickness.	In vitro	[257]
18	*Cymbopogon citratus* (DC.) Stapf	Whole plant	Terpenoids	Antimicrobial activity.	In vitro	[258]
19	*Cyperus rotundus* L.	Rhizome	Terpenoids	Antibacterial activity.	In vitro	[259]
20	*Dendrobium officinale* Kimura et Migo	Rhizome	Polysaccharides	The mitigation of UPEC-promoted pyroptosis in macrophage cells; the inhibition of the NLRP3/caspase-1/GSDMD pathway and ROS signal activation.	In vitro	[260]
21	*Equisetum arvense* L.	Leaves	Phenolics	Antimicrobial activity; the inhibition of biofilm mass production; antiadhesive action; modifications to the bacterial surface structures responsible for binding to the occupied surface.	In vitro	[235,246]
22	*Galium odoratum* (L.) Scop.	Leaves	Phenolics	Modifications to the bacterial surface structures responsible for binding to the occupied surface.	In vitro	[246]
23	*Gynostemma pentaphyllum* (Thunb.) Makino	Leaves	Terpenoids, dammarane-type saponins	Reduction in pro-inflammatory response of BECs to UPEC; the modulation of antimicrobial peptides; NF-κB inhibition and ERK activation.	In vitro	[261]
24	*Herniaria glabra* Linnaeus.	Leaves	Phenolics	High bactericidal activity; the inhibition of biofilm mass production; modifications to the bacterial surface structures responsible for binding to the occupied surface.	In vitro	[246]
25	*Labisia pumila var. alata* (Scheff.) Mez	Herbal	Phenolics	Reduction in uroepithelial apoptosis and the number of intracellular UPEC cells in BECs; reduction in the expression of β1 integrin	In vitro	[262]
26	*Lactuca indica* L.	Leaves	Terpenoids, phenolics	Reduction in the bacterial colonization of bladder epithelial cells; the inhibition of FAK, significantly decreasing bacterial adherence.	In vitro	[263,264]
27	*Lawsonia inermis* L.	Leaves	Xanthones	Antimicrobial activity.	In vitro	[249]
28	*Ocimum gratissimum* L.	Leaves, flowers	Terpenoids	Antimicrobial activity.	In vitro	[258]
29	*Orthosiphon stamineus* Benth.	Leaves	Flavonoids, terpenoids, essential oils	Antiadhesive effects; direct interaction between compounds from the extract and the bacterial adhesins.	In vivo	[235,265]
30	*Parkia biglobosa* (Jacq.) Benth	Roots, bark	Phenolics	Antibacterial activity.	In vitro	[266,267]
31	*Peganum. Harmala* L.	Seeds	Alkaloids, quinazoline derivatives	Antibacterial Activity.	In vitro	[268,269]
32	*Petasites albus* (L.) Gaertn.	Leaves, flower stems, rhizomes	Terpenoids	Biofilm-modulating activity on UPEC.	In vitro	[229,270]
33	*Petasites hybridus* (L.) G.Gaertn., B.Mey. and Schreb.	Leaves, flower stems, rhizomes	Terpenoids	Biofilm-modulating activity on UPEC.	In vitro	[229,270]
34	*Petroselinum crispum* (Mill.) Hill	Leaves	Phenolics	Antibacterial activity.	In vitro	[271]
35	*Piper arboreum* Aubl.	Leaves	Terpenoids	Modulatory activity, synergistic activity with antibiotic drugs.	In vitro	[272,273]
36	*Persicaria capitata* (Buch.-Ham. ex D. Don) H. Gross	Whole plant	Terpenoids, phenolics	Anti-inflammatory and moderate antibacterial activity.	In vitro, in vivo	[274]
37	*Punica granatum* L.	Seed	Phenolics	Antibacterial activity.	In vitro	[259]
38	*Rhodiola rosea* L.	Roots, rhizomes	Phenolics	Biofilm-modulating activity on UPEC.	In vitro	[229]
39	*Rosa canina* L.	Fruit	Vitamins, minerals	The prevention of UTIs.	In vitro, clinical	[275]
40	*Rosmarinus officinalis* L.	Leaves	Phenolics	Antibacterial activity.	In vitro	[271]
41	*Salvia officinalis* L.	Leaves	Terpenoids	Antimicrobial activity.	In vitro	[258]
42	*Salvia plebeia* R. Br.	Whole plant	Flavonoids, terpenoids, Phenolic acids	Diuretic activity; UPEC susceptibility.	In vitro, in vivo	[276,277]
43	*Schefflera leucantha* R. Viguier	Leaves	terpenoids, saponins	Antibacterial activity.	In vitro	[278,279]
44	*Toddalia asiatica* (L.) Lam.	Whole plant, leaves	Phenolics alkaloids	Antibacterial activity against MDR UPEC.	In vitro	[241,280]
45	*Tropaeoli majoris* herba	Leaves	Isothiocyanates	Intermediate susceptibility; possible damage to the cell membrane.	In vitro, in vivo, clinical	[243,244]
46	*Urtica dioica* L.	Leaves	Phenolics	Antimicrobial activity; antiadhesive effects; modifications to the bacterial surface structures responsible for binding to the occupied surface, and the direct interaction between compounds from the extract and the bacterial adhesins.	In vitro, in vivo	[235,246]
47	*Vaccaria segetalis* (Neck.) Garcke	Seeds	Polysaccharides	The upregulation of innate immunity in the kidney.	In vivo	[281]
48	*Vaccinium macrocarpon* Aiton	Fruit	polyphenols	The obstruction of bacterial adhesion to bladder cells; downregulation or interference with several bacterial virulence factors. The repression of the inflammatory cascades triggered by the immune system; the inhibition of UPEC motility.	In vitro, in vivo	[218]
49	*Vaccinium vitis-idaea* L.	Leaves	Phenolics	High bactericidal activity; the inhibition of biofilm mass production; modifications to the bacterial surface structures responsible for binding to the occupied surface.	In vitro	[246]
50	*Vernonia amygdalina* L.	Leaves stems	Terpenoids	Antimicrobial activity.	In vitro	[249,282]
51	*Zea mays* L.	Stigmata	Phenolics	Decreased bacterial adhesion; interaction with bacterial outer-membrane proteins.	In vivo, in vitro	[235]
52	*Zingiber officinale* Roscoe	Rhizomes	Terpenoids	Antibacterial activity.	In vitro	[283]

## Data Availability

No new data were created or analyzed in this study. Data sharing is not applicable to this article.
